# Imaging of a Rare Foreign Body Within the Female Pelvis Following Recent Laparoscopic Hysterectomy: A Case Report

**DOI:** 10.7759/cureus.80096

**Published:** 2025-03-05

**Authors:** Caroline Tomanek, Anna Eshghi, Andrew Buchan, Matthew L Roberts, Naghmeh Eshghi

**Affiliations:** 1 Department of Radiology, University of Arkansas for Medical Sciences, Little Rock, USA; 2 Department of Surgery, University of Arkansas for Medical Sciences, Little Rock, USA

**Keywords:** abscess, ct pelvis, foreign bodies, hysterectomy, pelvis imaging, vaginal dehiscence

## Abstract

Foreign bodies are commonly encountered in the female pelvis and may be placed intentionally or unintentionally due to underlying pathological conditions. It is imperative for radiologists to accurately identify these foreign objects to guide appropriate clinical care. In this case, a retained condom was misinterpreted as an abscess in a 45-year-old patient following a total hysterectomy complicated by vaginal cuff dehiscence and a previous repair at an outside hospital. This case highlights the importance of obtaining a detailed clinical history in the identification of foreign bodies, assessing potential complications, and guiding further management.

## Introduction

Detecting foreign bodies is a diagnostic challenge for radiologists in daily practice. Depending on the material and density of the foreign body, imaging features vary across different imaging modalities. Ultrasound can be considered for detecting and localizing radiolucent materials such as wood and plastic, although CT is more reliable for identifying radiopaque objects such as metal, stone, and glass [1]. Furthermore, as described by Tseng et al., foreign bodies can be accurately identified when correlated with clinical history and key imaging features [2]. Foreign bodies, whether intentional, unintentional, or post-surgical, can be found within the abdomen and pelvis. However, their varying densities present unique imaging characteristics that challenge radiologists [3]. Unintentionally retained foreign objects after abdominal surgery can trigger an acute inflammatory response leading to abdominal pain, abscess, or fistula formation, or, over time, may result in chronic inflammation with fibrosis [4]. Vaginal dehiscence is a rare complication that can occur after hysterectomy. Eoh et al. reported an incidence of 0.81% among patients who underwent hysterectomy [5]. Risk factors for vaginal dehiscence vary and depend on multiple factors such as surgical approach, wound healing, and mechanical influences such as early resumption of sexual activity and increased abdominal pressure [6,7]. We present the case of a 45-year-old woman who underwent a total laparoscopic hysterectomy, complicated by multiple episodes of vaginal dehiscence. Following early resumption of sexual activity, a retained condom within the pelvis was misinterpreted as an abscess due to the lack of clinical information.

## Case presentation

**Figure 1:**
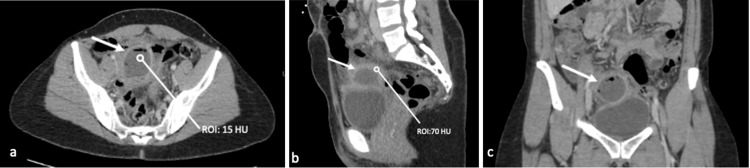
CT abdomen pelvis with IV contrast CT abdomen and pelvis with contrast axial (a), sagittal (b), and coronal (c) images demonstrate a thick-walled fluid-like collection (white arrow) containing gas superior to the bladder with an internal density of 15 HU and rim density of 70 HU.

A 45-year-old female with a history of total laparoscopic hysterectomy with bilateral salpingo-oophorectomy, complicated by vaginal cuff dehiscence and a recent repair, presented to the emergency department with abdominal pain, fever, and chills. Physical examination was notable for suprapubic abdominal tenderness. Laboratory results were remarkable for an elevated white blood cell count of 11.4 (reference range: 3.60-9.50). A contrast-enhanced CT of the pelvis was obtained, revealing a thick-walled fluid-like collection within the pelvis, superior to the bladder dome, with surrounding phlegmonous changes (Figure 1).

**Figure 2:**
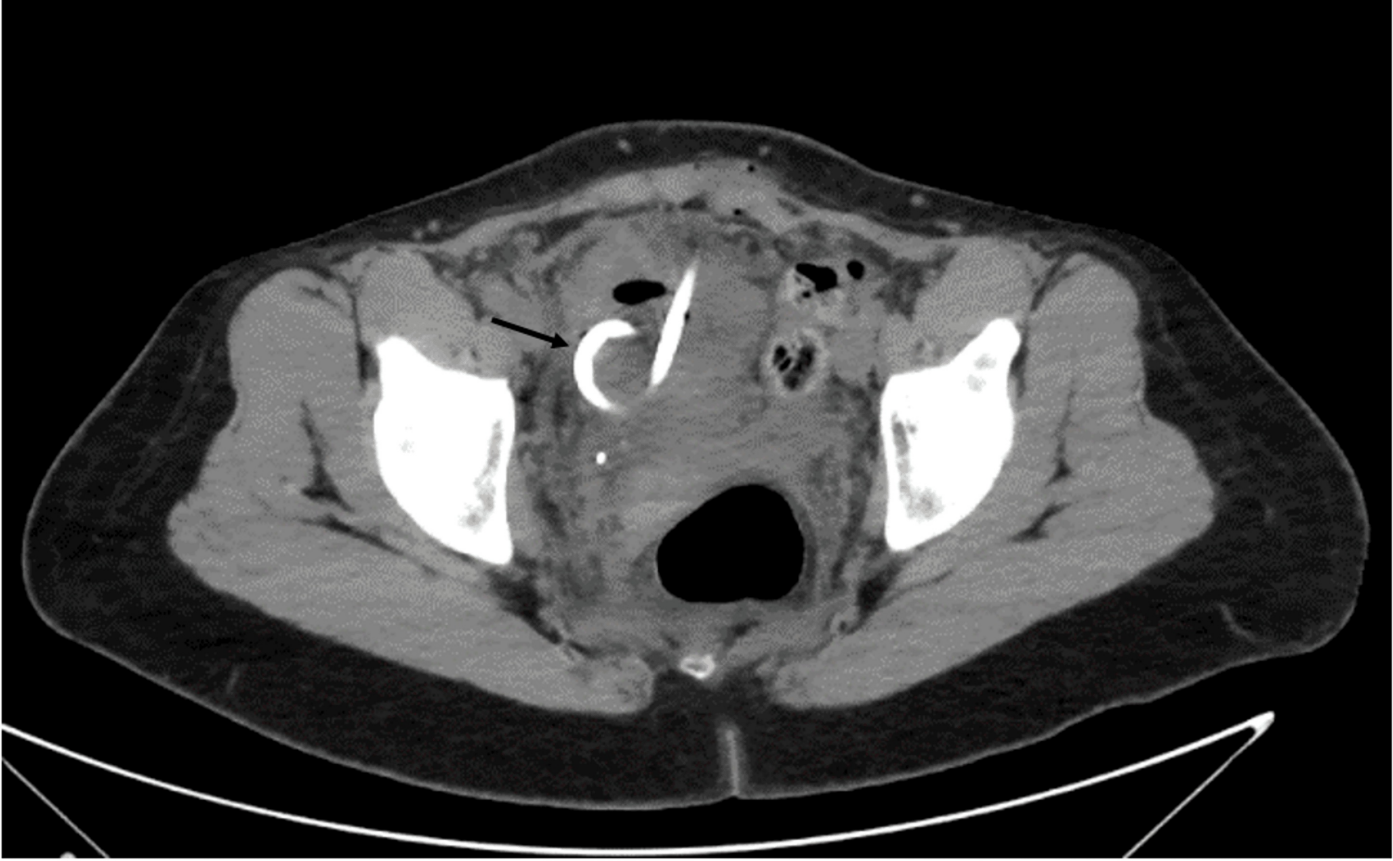
CT image during pigtail catheter placement CT axial images of the pelvis during pigtail catheter placement (black arrow) shows that the  fluid-like collection collapsed.

The fluid-like collection exhibited an internal density of 15 Hounsfield units (HU) and a rim-thick wall with a density of 70 HU. This finding was interpreted as a post-surgical intrapelvic abscess (Figure 1). The patient was admitted for intravenous antibiotic treatment and possible drain placement. However, CT-guided drainage of the fluid-like collection failed to aspirate pus or fluid and resulted in a collapse of the collection following the procedure (Figure 2).

**Figure 3:**
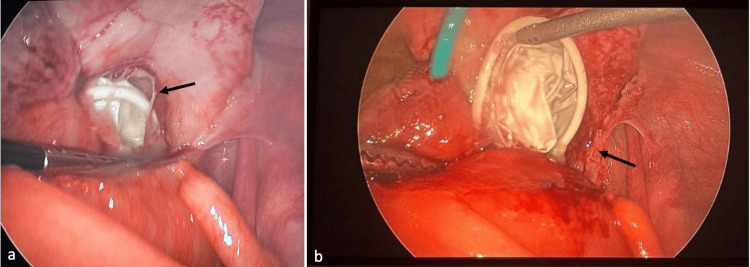
Diagnostic laparoscopic images Intraoperative images from laparoscopy demonstrate a foreign body with surrounding fibrotic and phlegmonous changes (black arrows), confirmed to be a retained condom.

Consultation with the clinician and reevaluation of the patient’s history revealed a recent laparoscopic hysterectomy, complicated by rare vaginal cuff dehiscence, which was repaired a few weeks prior. The patient admitted to having intercourse one week ago and was unable to locate the condom afterward. At that time, a pelvic exam was performed; however, the condom was not found, but vaginal cuff dehiscence was identified. Subsequent diagnostic laparoscopy revealed a condom within the right pelvis, with evidence of surrounding infection, phlegmonous changes, and small bowel adhesions (Figure 3).

The pigtail catheter and condom were subsequently removed, and the surrounding abscess cavity was drained. The patient was discharged home two days later with a course of oral broad-spectrum antibiotics. 

## Discussion

Intentionally or unintentionally retained foreign bodies are commonly encountered in the emergency department, and radiology is heavily relied upon for both initial and follow-up imaging [2]. Intentionally placed foreign bodies in the abdomen and pelvis include intrauterine devices, vaginal rings, and vaginal pessaries, which exhibit specific imaging features depending on their material and density on CT and multidetector CT [3,8]. Utilizing the correct imaging modality and recognizing the distinctive imaging features of foreign objects are crucial for prompt identification, diagnosis, and treatment. The imaging characteristics of foreign bodies vary across radiographs, CT, and ultrasound, depending on their material and density [1,2]. Ultrasonography is an accurate and reliable method for detecting radiolucent foreign bodies [1]. However, radiography and CT are frequently used to detect radiopaque objects. CT can also aid in evaluating radiolucent foreign bodies by assessing HU as a standardized CT density measurement and identifying complications related to retained objects [2,9].

In an uncomplicated post-hysterectomy CT scan, one would expect to see the absence of the uterus with a smooth vaginal cuff. However, in this case, CT of the pelvis revealed a fluid-like collection above the vaginal cuff with an internal density of 15 HU and a thick-walled density of 70 HU. These findings mimicked an abscess rather than a foreign object. Furthermore, the pertinent clinical history of vaginal dehiscence following laparoscopic hysterectomy, subsequent repair, early presumed intercourse, and a missing condom was not available at the time of imaging interpretation. Misinterpretation of the CT findings as abscess formation, followed by an unnecessary invasive procedure, resulted in a delay in clinical treatment. As Wang et al. described, a delay in treatment can increase patient morbidity and mortality due to potential complications such as infection, abscess or fistula formation, and sepsis [4]. In suspected retained foreign bodies in an emergency setting, collaboration between the clinician and radiologist, along with familiarity with the imaging characteristics of foreign bodies, is crucial for prompt diagnosis and treatment [8].

## Conclusions

In conclusion, radiology plays an important role in detecting and subsequently diagnosing foreign bodies by utilizing different imaging modalities. In addition to knowing key imaging features, including the density and shape of foreign bodies, clinical history is essential for accurate diagnosis and treatment. 
